# The Experiences of Autistic Adults Who Were Previously Identified as Having BPD/EUPD: A Phenomenological Study

**DOI:** 10.1192/bjo.2024.256

**Published:** 2024-08-01

**Authors:** Bruce Tamilson, Sebastian Shaw, Jessica Eccles

**Affiliations:** Brighton and Sussex Medical School, Brighton, United Kingdom

## Abstract

**Aims:**

This study aims to explore the experiences of autistic adults who were previously diagnosed with Borderline Personality Disorder (BPD).

**Methods:**

This interpretive phenomenological study aims to explore the experiences of autistic adults who were previously diagnosed with BPD. Data were collected using sixty-minute, one-to-one, virtual, semi-structured interviews. The audio-recordings of the interviews were transcribed and analysed using an interpretive phenomenological analysis.

**Results:**

Participants had autistic features since childhood which went unnoticed. Camouflaging, gender and lack of awareness of the spectrum nature of autism had contributed to missing autism in childhood. The commonality of trauma, suicidality and self-harm, in the context of wider systemic issues, resulted in participants receiving a diagnosis of BPD. It was revealed that the diagnosis of BPD was readily given and inappropriately disclosed. This diagnosis was emotionally damaging for participants and highly stigmatising. Treatment for BPD was inadequate, ineffective, and distressing. There were several negative impacts of the BPD label, including diagnostic overshadowing. Participants felt that misdiagnosis is preventable with various measures. Autism diagnoses were difficult to obtain in adulthood, but receipt of one was beneficial for participants in various ways. However, participants felt there was a need for more autism awareness and autism-friendly services.

**Conclusion:**

The BPD label in autistic people can be harmful to their physical, mental and social health. In contrast, an autism diagnosis in adulthood can be beneficial despite the multiple barriers in receiving such diagnosis. Misdiagnosis is preventable by training clinicians, screening risk groups and developing dedicated autism services.

